# Larynxorganerhalt bis zum T4‑Larynxkarzinom?

**DOI:** 10.1007/s00106-022-01180-y

**Published:** 2022-05-24

**Authors:** Gerhard Dyckhoff, Rolf Warta, Christel Herold-Mende, Peter K. Plinkert, Heribert Ramroth

**Affiliations:** 1 Universitäts-Hals-Nasen-Ohrenklinik Heidelberg, Im Neuenheimer Feld 400, 69120 Heidelberg, Deutschland; 2 Neurochirurgische Universitätsklinik Heidelberg, Im Neuenheimer Feld 400, 69120 Heidelberg, Deutschland; 3 Heidelberger Institut für Global Health, Universität Heidelberg, Gebäude 6130.3, Ebene 6, Im Neuenheimer Feld 130.3, 69120 Heidelberg, Deutschland

**Keywords:** Kehlkopfkrebs, Organerhalt, Radiochemotherapie, Totale Laryngektomie, Überleben, Laryngeal carcinoma, Organ preservation, Radiotherapy, Chemoradiotherapy, Survival

## Abstract

Kann die primäre Radiochemotherapie (pRCT) möglicherweise als alternative Standardtherapie zur totalen Laryngektomie (TL) angesehen werden? Gemäß der neuen S3-Leitlinie nehme der Patient zwar eine höhere Rückfallrate in Kauf, habe aber die Salvagechirurgie als kurative Option und insgesamt keinen Überlebensnachteil. In mehreren großen Datenbankanalysen und Fallserien findet sich für das T4-Larynxkarzinom ein signifikanter Überlebensnachteil der pRCT gegenüber der primären TL von über 30 %. Die Erfolgsrate der Salvagelaryngektomie beim T4-Karzinom liegt laut Literatur nur bei 25–50 %. Larynxorganerhaltstudien, die zur Empfehlung der pRCT als alternativer Standardtherapie führen könnten, sollten 1.) T4a-Karzinompatienten innerhalb ihrer T‑Kategorie auswerten, 2.) getrennt nach Larynx- und Hypopharynxkarzinomen, 3.) in aussagekräftiger Kollektivgröße, 4.) mit einem Nachbeobachtungszeitraum von mindestens 5 Jahren, 5.) mit onkologischen und 6.) funktionellen Outcome (Dauer von Tracheostoma u./o. PEG, Notwendigkeit und Erfolg einer Salvagelaryngektomie). 7.) Angabe des Kriteriums, das zur T4-Kategorie führte (Knorpeldurchbruch oder Art der extralaryngealen Ausbreitung), 8.) prätherapeutische Larynxfunktionalität (Tracheostoma, PEG). Eine Erfassung der genannten klinischen Daten aller T4-pRCT-Patienten in einer prospektiven beobachtenden Kohortenstudie im deutschsprachigen Raum wird vorgeschlagen. Bei Ablehnung der TL beim T4a-Karzinom sollte in ausführlichen einfühlsamen, aber instruktiven Gesprächen unterschieden werden zwischen einer primären spontanen Abneigung und der differenziert abgewogenen, definitiven Ablehnung. Nicht nur der onkologische, sondern auch der funktionell zu erwartende Outcome sollte in den Entscheidungsprozess einbezogen werden.

## Abstract (en)

Could primary chemoradiotherapy (pCRT) possibly be viewed as an alternative standard therapy to upfront total laryngectomy (TL)? According to the new German S3 guideline, despite higher rates of local recurrence, there would be no survival disadvantage and salvage surgery would be a curative option. In several large database studies and case series, statistically significant survival disadvantages of more than 30% between pCRT and TL have been reported for T4 laryngeal cancer. According to the literature, the success rate of salvage TL for T4 laryngeal cancer is only about 25-50%. Larynx preservation (LP) studies which could qualify the recommendation of pCRT as an alternative standard therapy to TL in T4 carcinomas should 1) evaluate T4a cancers within the T4 category; 2) perform subgroup analysis of laryngeal and hypopharyngeal cancers; 3) be sufficiently highly powered; 4) provide long-term outcomes of at least 5 years; 5) with oncological and 6) functional outcomes (duration of the need for tracheostomy and/or feeding tube dependency; necessity and success of salvage laryngectomies). 7) Specification of the criteria of the respective T4 classification (invasion through the outer cortex of the cartilage, or infiltration of which extralaryngeal structures) and 8) evaluation of pretreatment laryngeal function (at least: tracheostomy, feeding tube dependency). Collection of all the aforementioned data of T4 patients treated with pCRT in a large prospective observational cohort study in German-speaking countries is suggested. In case of rejection of TL by T4 laryngeal cancer patients, differentiation between primary spontaneous reluctance and a definitive, carefully considered decision is important. This distinction should be achieved by sensitive discussions. Not only oncological but also functional outcome probabilities should be included in the overall decision-making process.

## Hintergrund und Fragestellung

In der neuen S3-Leitlinie zur Behandlung des Kehlkopfkarzinoms wird für Karzinome im Stadium cT4a cN0–cN3 ein primär chirurgisches Vorgehen, d. h. die totale Laryngektomie (TL), empfohlen, wenn eine R0-Resektion möglich erscheint [2]. Alternativ könne unter Inkaufnahme einer höheren lokalen Rückfallrate eine primäre Radiochemotherapie (pRCT) oder andere konservative Therapieverfahren durchgeführt werden [2]. Für multimodale Organerhaltungsprotokolle eigneten sich prinzipiell Patienten mit Glottiskarzinomen im Stadium T3–T4a, supraglottischen Karzinomen im Stadium T2–T4a und Hypopharynxkarzinomen im Stadium T2–T4 [2]. Unter Experten herrsche Uneinigkeit darüber, ob solche Therapiekonzepte weiterhin nur in Studien durchgeführt werden sollten oder aber zur alternativen Standardtherapie erklärt werden könnten. Begründung: hohe Ansprechrate bei adäquater Patientenselektion, Salvagechirurgie als kurative Option, fehlender Überlebensnachteil [2].

## Kein Überlebensnachteil nach pRCT beim T4-Larynxkarzinom?

Ist nach einer pRCT bei einem T4-Larynxkarzinom nur eine höhere Rückfallrate zu erwarten, oder ist auch mit einem Überlebensnachteil zu rechnen? Grover berichtet über den Outcome von 969 T4a-Patienten. Das mediane Überleben der TL-Patienten lag bei 61 Monaten gegenüber 39 Monaten bei den pRCT-Patienten. Allerdings bestand ein Selektionsbias: bei den konservativ Behandelten fand sich ein größerer Anteil an N2/3-Patienten (36,4 % gegenüber 24,3 %). Nach Korrektur dieses Bias in der multivariaten Analyse lag der Überlebensnachteil jedoch immer noch bei 30 %. Neben dieser einen Datenbankanalyse gibt es eine Vielzahl weiterer Studien, die übereinstimmend einen signifikanten Überlebensnachteil nach konservativer Organerhalttherapie im Vergleich zur primären TL mit adjuvanter Radiotherapie (aRT) beschreiben. Die ersten Studien wiesen dabei noch Verzerrungen zuungunsten des konservativen Ansatzes auf (s. Anmerkungen zu den Beispielen in Tab. 1).

**Table 1:** 

Studie (Behandlungszeitraum)	Therapiearme (Anzahl Patienten)	Ergebnisse/univariat	Signifikanzniveau/multivariat
Chen, 2007 [3] (1995–1998)	TL (*n* = 1690) RCT (358) RT (566)	k. A. (Vor 2003 keine Unterscheidung T4a/T4b, Einschlussbias: Inoperable eher R(C)T! Insgesamt relativ wenige RCT-Patienten!)	HR: 1,59 *p* < 0,001
Grover, 2015 [9] (2003–2006)	pRCT (*n* = 616) TL±aR(C)T (*n* = 353)	Medianes Überleben: 61 Mon. vs. 39 Mon. (Nur T4a, aber Bias bzgl. N2/3!)	HR: 1,31 (1,1–1,57) *p* < 0,001
Megwalu, 2014 [16] (1992–2009)	OP (*n* = 1737) pR(C)T (*n* = 1410)	5‑J.-DSS: 69 % vs. 56 % 5‑J.-OS: 56 % vs. 38 %	*p* < 0,001 *p* < 0,001
Dziegielewski, 2012 [6] (1998–2008)	TL±a(C)RT (*n* = 116) pR(C)T (*n* = 60) pRT (*n* = 82)	5‑J.-OS: 49 % vs. 16 % vs. 5 % 5‑J.-DFS: 45 % vs. 9 % vs. 3 %	HR^a^: 2,3 (1,0–5,0) HR^a^: 2,6 (1,2–5,5)
Timmermans, 2015 [24] (1991–2010)	TL±aR(C)T (*n* = 848) pRCT (*n* = 123) pRT (*n* = 560)	5‑J.-OS: 48 % vs. 42 % vs. 34 %	*p* < 0,0001
Stokes, 2017 [23] (2004–2012)	TL±aR(C)T (*n* = 1559) cRCT (*n* = 1597) iRCT (n.a., s. [5])	Medianes Überleben: 60 Mon. vs. 32 Mon.	HR: 1,55 (1,41–1,70)
Karatzanis, 2014 [13] (1980–2007)	TL+a(C)RT (*n* = 321) pR(C)T (*n* = 63)	5‑J.-OS: 41 % vs. 17 %	*p* < 0,001
Vengalil, 2016 [25] (2003–2010)	TL (*n* = 42) pR(C)T (*n* = 65)	3‑J.-OS: 70 % vs. 41 %	*p* < 0,01
Bates, 2019 [1] (2004–2015)	TL+aR(C)T (*n* = 1541) pRCT (*n* = 1900)	5‑J.-OS: 48,9 % vs. 39,4 %	HR: 1,34 (1,2–1,5) *p* < 0,01

*pRT* primäre Radiotherapie, *pRCT* primäre Radiochemotherapie, *OP* Operation, *aR(C)T* adjuvante Strahlentherapie mit oder ohne Chemotherapie, *cRCT* konkomitante Radiochemotherapie, *iRCT* Induktions-Radiochemotherapie, *TL* totale Laryngektomie, *OS* Gesamtüberleben, *DFS* krankheitsfreies Überleben, *DSS* krankheitsspezifisches Überleben, *HR* Hazard Ratio (mit 95%-Konfidenzintervall); *k.* *A.* keine Angabe; *n.a.* nicht anwendbar

^a^ für TL±a(C)RT vs. pRCT

In der aktuellsten Studie kritisierten Bates et al., dass man bisherigen Datenbankanalysen nicht berücksichtigt habe, ob eine ausreichende hohe Strahlendosis appliziert worden sei. Daher untersuchten sie aus einem Kollektiv von 113.751 Larynxkarzinompatienten der Jahre 2004–2015 nur diejenigen T4a-Patienten, die mit einer pRCT mit einer kurativen Dosis von 70–80 Gy behandelt worden waren (*n* = 1900) im Vergleich zu solchen, die eine TL+aR[C]T von 60–80 Gy erhielten (*n* = 1541). Auch hier fand sich ein signifikanter Überlebensnachteil für die pRCT gegenüber der primären TL von im Schnitt über 30 %: für T4 N0 eine Risikoquote (HR) von 1,39 (*p* < 0,001), für T4 N+ ein HR von 1,22 (*p* = 0,001) [1].

Diese Studien sprechen dafür, dass für das T4a-Larynxkarzinom nach pRCT nicht nur eine höhere Rückfallrate, sondern auch ein signifikanter Überlebensnachteil im Vergleich zur primären TL besteht.

## Wie sicher ist die Salvagechirurgie beim T4-Larynxkarzinom?

Bietet die Salvagelaryngektomie im Fall des Versagens der pRCT eine ausreichend sichere kurative Option für Patienten mit einem T4-Larynxkarzinom?

In einer der drei maßgeblichen Larynxorganerhaltstudien, der RTOG 91-11-Studie von Forastière, wird die Erfolgsrate der Salvagelaryngektomie mit durchschnittlich 70 % angegeben. Allerdings hängt die Erfolgsrate vom T‑Stadium ab. Johansen berichtet von einer Erfolgsrate von 79 % für glottische Larynxkarzinome im Stadium T1a, 68 % für T2, 60 % für T3 und nur 44 % für T4 [12]. Bei Parsons lag die Erfolgsrate nur bei 25 % für T4-Tumoren im Vergleich zu 50 % bei anderen T‑Kategorien [17]. Bei einer Erfolgsrate über alle T‑Kategorien von 70 % wird die Erfolgsrate für die T4-Karzinome (nur 10 % des Forastière-Kollektivs!) zwischen 25 % und 50 % gelegen haben. In der TREMPLIN-Studie (Induktionschemotherapie [ICT], gefolgt von RCT mit Cisplatin oder RIT mit Cetuximab) konnte im RCT-Arm bei keinem der Therapieversager (0/8) eine erfolgreiche Salvagelaryngektomie durchgeführt werden [15]. Insofern ist die Salvagelaryngektomie auch beim T4 zwar potenziell eine kurative Option, bietet aber in diesem Stadium nicht die Sicherheit, die man sich im Fall des Therapieversagens wünschen würde. Dies spiegelt sich letztlich auch in dem signifikant schlechteren Überleben nach pRCT im Vergleich zur primären TL wider.

Nicht zu vernachlässigen ist auch die im Vergleich zur primären TL signifikant höhere Komplikationsrate der TL nach pRCT [11, 22]. In einer Metaanalyse von fast 3300 Patienten in 50 Studien lag die Gesamtkomplikationsrate nach Salvage-TL bei knapp 70 %. Führend waren dabei pharyngokutanen Fisteln mit knapp 30 %. Als weitere signifikant häufiger auftretende Komplikationen angeführt wurden u. a. Wundinfektionen, Dehiszenzen, Nekrosen, Blutungen, Stenosen und Dysphagie [11]. Eine Möglichkeit zur Reduktion der Inzidenz von pharyngokutanen Fisteln ist die Deckung mittels unbestrahltem Gewebe z. B. einem Pektoralis-major-Lappen [21]. Für die Salvage-TL bei rT4-Karzinomen wurden signifikant höhere Komplikationsraten beschrieben als für rT1-, rT2- oder rT3-Tumoren (*p* = 0,007) [19].

## Sollte man die pRCT als alternative Standardtherapie beim T4-Larynxkarzinom empfehlen?

Nach erfolgreichem Larynxorganerhalt ohne statistisch nachweisbaren Überlebensunterschied wurde nach den grundlegenden Larynxorganerhaltstudien [7, 14, 27] die pRCT bis hin zum T4-Karzinom empfohlen (ASCO-Leitlinie zum Larynxorganerhalt, 2006 [18]). Eine Reevaluation der T4-Patienten zeigte jedoch, dass nur wenige T4-Patienten eingeschlossen worden waren und diese einen deutlich schlechteren Outcome zeigten: 5,6-fach geringere Ansprechrate auf die Induktionschemotherapie, 7,1-fach schlechterer Organerhalt mit 56 % Salvagelaryngektomien in der T4-Gruppe vs. 29 % bei den T1–T3 (weitere Einzelheiten in [5]). Da alle Tumorkategorien T2–T4 gemeinsam ausgewertet worden waren, wurde diese schlechtere Überleben der T4-Karzinom-Patienten statistisch zunächst nicht erkannt: Es wurde von dem relativ guten Ansprechen der zahlreichen, weniger weit fortgeschrittener Tumoren überdeckt. „Die wenigen Birnen fielen bei der Vielzahl der Äpfel nicht auf.“ Erst die Subgruppenanalyse der T4-Karzinom-Patienten brachte das signifikant schlechtere Überleben zum Vorschein [5]. Um eine verlässliche Aussage über das Outcome des T4-Larynxkarzinoms machen zu können, muss man es *innerhalb derselben T‑Kategorie* auswerten, man muss also „Birnen mit Birnen vergleichen“.

In der aktualisierten ASCO-Leitlinie zum Larynxorganerhalt von 2018 ist die ursprüngliche Empfehlung der pRCT bis zum T4 aufgrund der gegebenen Datenlage korrigiert worden. Jetzt heißt es, dass für ausgewählte Patienten mit ausgedehntem T3- oder T4a-Karzinom und/oder bereits vor Therapiebeginn eingeschränkter Kehlkopffunktion „möglicherweise bessere Überlebensraten und eine bessere Lebensqualität“ erreicht werden können, wenn eine totale Laryngektomie anstelle eines Organerhaltansatzes durchgeführt wird und dass dieser Ansatz „möglicherweise zu bevorzugen“ sei [8].

In der deutschen S3-Leitlinie zur Behandlung des Larynxkarzinoms wird maßgeblich die DeLOS-II-Studie zitiert [2]. Mit Einschluss eines Taxans in die Induktion und Bewertung des Frühansprechens mit einem neu entwickelten Laryngectomy-Free-Survival-Score konnten in einem Kollektiv von 173 Larynx- und Hypopharynxkarzinomen im Stadium T2–T4a (32 % T4a-Tumoren) sehr erfreuliche 2‑Jahres-Überlebensraten von ca. 69 % erzielt werden, sodass Wichmann et al. schlussfolgerten, dass T4a-Karzinome nicht grundsätzlich von Larynxorganerhaltstudien ausgeschlossen werden sollten [26]. Tatsächlich wäre es eine erfreuliche neue Option, wenn sich die Ergebnisse im Langzeitverlauf und weiteren Studien bestätigen. Zu beachten ist, dass laut Protokoll nur kleinvolumige T4-Tumoren eingeschlossen wurden; dabei durfte der Larynxknorpel infiltriert, aber nicht nach außen durchbrochen sein [2]. Ähnlich fand Vengalil für eine kleine Untergruppe so genannter günstiger („favorable“) kleinvolumiger T4a-Karzinome mit nur geringer Knorpelbeteiligung nach pRCT ein mit der primären TL vergleichbares 3‑Jahres-Überleben von 70 %, wobei hier Cisplatin als Chemotherapie verwendet wurde [25]. Die Gesamtgruppe der pRCT-Patienten zeigte jedoch eine signifikant schlechtere 3‑Jahres-Überlebensrate von nur 41 % vs. 70 % nach TL [25]. Eine Knorpelinfiltration ohne Knorpeldurchbruch ist jedoch nach geltender TNM-Klassifikation (UICC und AJCC) nur ein T3-Kriterium. Es stellt sich daher die Frage, ob bei den genannten „günstigen T4“ unabhängig von der Knorpelbeteiligung auch ein anderes T4-Kriterium vorgelegen hat, d. h. eine extralaryngeale Ausbreitung, z. B. in die Trachea, den Ösophagus oder die äußere Zungenmuskulatur. Dziegielewski weist darauf hin, dass Tumoren ohne Knorpeldurchbruch nach heutigem Standard als „T3 downgestaged“ würden [6]. Sanabria bezeichnet die Beschränkung per Studienprotokoll auf „minimale Schildknorpelinvasion oder (lediglich) Verdacht auf Invasion in der Bildgebung“ als „Selektionsbias“ und warnt davor, Ergebnisse solcher randomisierter kontrollierter Studien (CRS) zum Kehlkopferhalt auf das Standardvorgehen zu übertragen [20]. Zudem ist ein *ausreichend langer Nachbeobachtungszeitraum* erforderlich, denn es gilt nicht nur das kurzfristige Ansprechen zu beurteilen, sondern den langfristigen Therapieerfolg. Die DeLOS-II-Studie rekrutierte zwischen 2007 und 2012. Als „final results“ wurden 2018 aber bisher erst 2‑Jahres-Überlebensdaten präsentiert [4]. Da aber gerade bei erhaltenem Kehlkopf auch nach mehr als 5 Jahren aus verbliebenen ruhenden Tumorzellen noch In-Field-Rezidive auftreten können, wäre es wesentlich, Langzeitergebnisse abzuwarten. Zur Validierung der Ergebnisse der DeLOS-II-Studie wäre daher ein Update der Überlebensdaten zielführend, mit zumindest deskriptiver Subgruppenanalyse der T4-Tumor-Patienten.

Forastière stellte fest, dass ein statistischer Vergleich bei den T4-Tumoren der RTOG-91-11-Studie nicht möglich gewesen sei, da nur 10 % der in der eingeschlossenen Studie T4-Karzinome aufwiesen; in ihrer Studie waren dies insgesamt 51 Patienten mit T4-Karzinom. Der Aufwand einer randomisierten kontrollierten Studie, den die Kollegen der DeLOS-II-Studie geleistet haben, ist immens und wird kaum für ein ausreichend großes Kollektiv an T4-Larynxkarzinomen umsetzbar sein. Unsere regelmäßige Erfahrung ist jedoch, dass immer wieder Patienten eine nach der offiziellen Empfehlung der aktuellen S3-Leitlinie beim cT4a cN0–cN3 indizierte Laryngektomie trotz aller Beratung ablehnen und eine pRCT vorziehen. Um in der Zukunft ein *ausreichend großes Kollektiv* an T4-Larynxkarzinomen zur Auswertung zur Verfügung zu haben, könnten wir über unsere Fachgesellschaft die Daten dieser Patienten, die in den verschiedensten Kliniken im deutschsprachigen Raum mittels pRCT behandelt werden, sammeln und im Sinne eine beobachtenden Kohortenstudie auswerten. Biologie und Prognose der Tumoren der unterschiedlichen Lokalisationen sind sehr unterschiedlich. Daher wäre es sinnvoll, nach dem „Äpfel-zu-Äpfeln-Prinzip“ nach der *Lokalisation* zu unterscheiden (glottisches Larynxkarzinom, supraglottisches Larynxkarzinom, Hypopharynxkarzinom) und zusätzlich nach der *Art der Therapie* (Induktion z. B. nach Maßgabe der DeLOS-II oder simultane cisplatinbasierte pRCT). Für die internationale Vergleichbarkeit wäre es hilfreich, bei der Charakterisierung der Patienten anzugeben, welches Kriterium zur Klassifizierung als T4-Kategorie geführt hat (Knorpel*durchbruch, Art der extralaryngealen Ausbreitung*). Um den Einfluss der *laryngealen Funktionsstörung* auf das onkologische und funktionelle Outcome zu erfassen, sollte zusätzlich erfasst werden, ob vor Therapiebeginn bereits eine Tracheotomie erforderlich war oder eine PEG-Abhängigkeit bestand. In gleicher Weise sollte beim *Outcome* nicht nur das *Überleben* erfasst werden, sondern auch die *Funktion*, als Mindestabfrage die posttherapeutische Dauer von Tracheotomie und/oder PEG-Versorgung und im Bedarfsfall, ob eine *Salvage-TL* erforderlich wurde und ob diese *erfolgreich* war. Die Aussagekraft einer solchen Beobachtungsstudie wäre deutlich geringer als diejenige einer kontrollierten randomisierten Studie (CRS). Aber ein gewisser Anhalt für das Outcome von pRCT unter allgemeinen Klinikbedingungen könnte an einem großen Patientenkollektiv gewonnen werden und Grundlage für die Beratung von Patienten werden, die unter genau diesen allgemeinen Klinikbedingungen außerhalb von CRS behandelt werden.

## Beratung des Patienten

Kaum ein Patient wird bei der Eröffnung der onkologisch grundsätzlich gebotenen Notwendigkeit einer totalen Laryngektomie mit Begeisterung zustimmen. Daher ist es wesentlich, eine primäre spontane Abneigung gegen die TL von einer gut abgewogenen definitiven Ablehnung zu unterscheiden. Hierzu bedarf es der offenen Information über die verschiedenen Behandlungsmöglichkeiten einschließlich der zu erwartenden onkologischen und funktionellen Erfolgsaussichten, abhängig von den individuellen Risikofaktoren. Die TL und die pRCT beeinträchtigen verschiedene Funktionsaspekte unterschiedlich, wobei interessanterweise die Gesamtlebensqualität nach beiden Therapieverfahren z. T. sogar als vergleichbar eingeschätzt wird [10]. Daher sollten die Folgen und Risiken beider Optionen detailliert besprochen werden, um dem Patienten ein umfassendes Bild zu vermitteln. Ganz wesentlich ist eine Darstellung der verschiedenen Möglichkeiten der Stimmrehabilitation, bei der auch Logopäden und Mitglieder von Selbsthilfeorganisationen zu Wort kommen sollten. Diese Gespräche sollten nach Möglichkeit gemeinsam mit Familienangehörigen und/oder vertrauten Freunden erfolgen, um das soziale Umfeld mit einzubeziehen. Nicht zuletzt sollten die Präferenzen des Patienten beachtet werden und seine Risikobereitschaft, für eine möglicherweise bessere Lebensqualität ggf. eine eventuell geringere Überlebenswahrscheinlichkeit in Kauf zu nehmen. Hierbei sollten die Aussichten nicht rein nach der formalen T‑Klassifikation besprochen werden, sondern ganz differenziert nach den individuellen Gegebenheiten. So kann man einem Patienten mit einem kleinen Hypopharynxkarzinom der medialen Wand des Sinus piriformis mit minimaler Arrosion des Schildknorpels (beim Hypopharynxkarzinom ein T4-Kriterium!), aber noch völlig unbeeinträchtigter Schluck- und Sprechfunktion trotz formal schlechterer Prognose bei Ablehnung der Laryngektomie den Versuch des Larynxerhalts mittels pRCT anbieten. Hingegen wird man einem Patienten selbst bei einem glottischen Larynxkarzinom „nur“ im Stadium T3 eher zur Laryngektomie raten, wenn der Schildknorpel großflächig infiltriert ist, der Patient bereits nottracheotomiert werden musste und zudem bei Niereninsuffizienz keine Kombination mit Cisplatin erhalten könnte. Gerade in solchen Fällen ist nicht nur der onkologische Outcome, sondern auch das zu erwartende funktionell ungünstige Ergebnis mit möglicherweise bleibendem Tracheostoma und/oder PEG-Sonden-Abhängigkeit trotz erhaltenem Kehlkopf zu besprechen, bis hin zur Notwendigkeit der funktionellen Laryngektomie trotz ggf. vollständiger Tumorfreiheit; denn Kehlkopferhalt ist nicht gleichbedeutend mit Funktionserhalt.

## Fazit für die Praxis

- Beim T4-Larynxkarzinom droht nach primärer Radiochemotherapie (pRCT) nicht nur ein höheres Rückfallrisiko, sondern die Überlebenswahrscheinlichkeit ist nach der aktuellen Datenlage signifikant schlechter als nach totaler Laryngektomie (TL).
- Bei Ablehnung der Laryngektomie beim T4-Karzinom sollte in ausführlichen einfühlsamen, aber instruktiven Gesprächen unterschieden werden zwischen einer primären spontanen Abneigung und der differenziert abgewogenen, definitiven Ablehnung.
- Hierbei sollten nicht nur der onkologische, sondern auch der funktionell zu erwartende Outcome in den Entscheidungsprozess einbezogen werden.
- Eine beobachtenden Kohortenstudie aller damit einverstandenen Patienten, die bei T4 die TL ablehnen und eine pRCT durchführen lassen, könnte ein aussagekräftiges Kollektiv von unter allgemeinen Klinikbedingungen behandelten Patienten ergeben, das für die künftige Beratung hilfreich sein dürfte.
